# Menopausal hormone therapy and incidence, mortality, and survival of breast cancer subtypes: a prospective cohort study

**DOI:** 10.1186/s13058-024-01897-4

**Published:** 2024-11-04

**Authors:** Marit Busund, Giske Ursin, Eiliv Lund, Sairah Lai Fa Chen, Charlotta Rylander

**Affiliations:** 1 Department of Community Medicine, Faculty of Health Sciences, UiT The Arctic University of Norway, 9037 Tromsø, Norway; 2 Cancer Registry of Norway, Norwegian Institute of Public Health, Oslo, Norway; 3 Institute of Basic Medical Sciences, University of Oslo, Oslo, Norway; 4 Department of Preventive Medicine, University of Southern California, Los Angeles, CA USA

**Keywords:** Menopausal hormone therapy, Breast cancer subtypes, Incidence, Mortality, Survival

## Abstract

**Background:**

Menopausal hormone therapy (MHT) is associated with an increased risk of postmenopausal breast cancer, predominantly the luminal A-like subtype. The impact of MHT on deaths from breast cancer subtypes is less understood. This study aimed to explore associations between MHT use and the incidence, mortality, and survival of intrinsic-like breast cancer subtypes.

**Methods:**

Data from 160,881 participants with self-reported MHT use from the prospective Norwegian Women and Cancer Study were analyzed. Among them, 7,844 incident breast cancer cases, and 721 breast cancer-specific deaths occurred. Cox proportional hazard regression was performed to calculate hazard ratios (HRs) with 95% confidence intervals (CIs) for the association between MHT use and the incidence, mortality, and survival of breast cancer subtypes.

**Results:**

MHT use was associated with increased risk of overall, luminal A-like, and luminal B-like breast cancer, with respective HRs of 1.44 (95% CI 1.36–1.52), 1.41 (95% CI 1.31–1.52), and 1.23 (95% CI 1.09–1.40) among current estrogen-progestin therapy (EPT) users compared with never users. The risk increased by 4%, 4%, and 2% per year of EPT use for overall, luminal A-like, and luminal B-like breast cancers, respectively. MHT use was also associated with increased risk of overall and luminal A-like breast cancer mortality, with HRs 1.61% (95% CI 1.36–1.91) and 2.15% (95% CI 1.51–3.05) increased risk among current EPT users compared with non-users. Among patients with breast cancer, pre-diagnostic MHT use was not associated with worse survival from overall breast cancer but was inversely associated with survival from triple-negative breast cancer (TNBC; HR death 0.41; 95% CI 0.24–0.73 among current users). Results varied significantly according to tumor subtype (*p*_*heterogeneity*_ = 0.02).

**Conclusions:**

Our study suggests that MHT use increases the risk of incident and fatal overall and luminal A-like, and incident luminal B-like breast cancer but does not decrease overall survival among patients with breast cancer. Further research is needed to elucidate the mechanisms underlying MHT use and breast cancer lethality, and to explore whether MHT use among patients with TNBC is indeed free from harm.

**Supplementary Information:**

The online version contains supplementary material available at 10.1186/s13058-024-01897-4.

## Background

Breast cancer is a heterogeneous disease with intrinsic molecular tumor subtypes that have different risk factors, tumor characteristics, responses to treatment, and survival outcomes [1–5]. These molecular subtypes are commonly cross-classified into a surrogate definition referred to as intrinsic-like subtypes using standard immunohistochemical (IHC) analyses of tumor receptor status [6].

Over the last three decades, numerous studies have identified combined menopausal hormone therapy (MHT) as an important risk factor for postmenopausal breast cancer [7–14]. The latest analyses by the Collaborative Group on Hormonal Factors in Breast Cancer found that all types and regimens of MHT, except vaginal estrogens, were associated with increased risk [13]. The risk escalated with longer use, with estrogen-progestin therapy (EPT) posing a higher risk than unopposed estrogen therapy (ET) compared with non-use [13]. Many studies have investigated the associations between MHT use and intrinsic-like subtypes of breast cancer. A uniform consensus that MHT use is associated with luminal A-like (estrogen receptor (ER)-positive/progesterone receptor (PR)-positive/human epidermal growth factor 2 (HER2)-negative) breast cancer is apparent [15–21], while some studies have indicated a similar association with luminal B-like (ER + /any PR/HER2 + or ER + /PR-/HER2-) subtypes [16, 19–21]. Indications of increased risks of hormone receptor-negative [22] and triple-negative breast cancer (ER-/PR-/HER2-; TNBC) [21] associated with MHT have also been reported, although findings regarding MHT use and hormone receptor-negative subtypes, including TNBC and HER2-enriched (ER-/PR-/HER2 +), are inconsistent.

Contrary to breast cancer incidence, evidence on the impact of MHT use on breast cancer-specific mortality and survival is conflicting. Numerous studies have been published [23–36]; however, the results have been ambiguous and, possibly, afflicted by collider stratification bias. Studies examining breast cancer-specific mortality among the entire study population have reported an increased risk associated with MHT use [23, 26]. Conversely, studies of patients with breast cancer have generally indicated improved survival among pre-diagnostic MHT users [24, 29–33]. A pooled analysis from the Breast Cancer Association Consortium (BCAC) with 121,435 breast cancer cases and 8,554 breast cancer-specific deaths also demonstrated improved survival among MHT users [29]. Studies evaluating the association between pre-diagnostic MHT use and breast cancer subtype-specific mortality and survival are sparse. However, the pooled BCAC analysis found increased survival across all subtypes with EPT and ET formulations [29].

While breast cancer mortality refers to the incidence of breast cancer deaths among initially healthy women, breast cancer survival measures the case-fatality among women diagnosed. Hence, mortality reflects the effects of both incidence and lethality, whereas survival specifically measures lethality and, consequently, more accurately assesses the impact of pre-diagnostic MHT on the developmental pathways of carcinogenesis that may influence tumor aggressiveness. However, survival can be influenced by several biases arising from early detection, typically through cancer screening or high awareness linked to socioeconomic status [37, 38].  These biases can obscure the understanding of cancer lethality. Thus, the importance of interpreting survival in the context of incidence and mortality has been emphasized [38, 39]. Increased knowledge of the relationship between MHT use and mortality and survival in breast cancer subtypes could be valuable for mitigating risks and prognostication for patients with breast cancer. This study aimed to investigate the associations between MHT use and the incidence, mortality, and survival of intrinsic-like breast cancer subtypes.

## Methods

### Study population

The Norwegian Women and Cancer (NOWAC) study [40], initiated in 1991, is a comprehensive, national prospective cohort study designed to explore cancer etiology in Norwegian women. Participants aged 30–70 years were randomly selected from the National Population Register between 1991 and 2008. A total of 172,472 women participated, completing up to three follow-up questionnaires approximately every 6 years. The unique national identification number for all Norwegian residents allows for complete follow-up through linkages to national registries [41]. The NOWAC study has demonstrated considerable external validity; the distribution of risk factors is independent of response rates, and cancer incidence rates align with national data from the Cancer Registry of Norway [42].

From the total cohort of 172,472, we excluded those with missing MHT status at the start of follow-up (n = 2,063), prevalent cancers (other than non-melanoma skin cancer; n = 7,862), premenopausal breast cancers (n = 1,004), participants who had died or emigrated before follow-up (n = 501), and those with extreme values for age at menarche (< 8 or > 20 years; n = 30), age at menopause (< 25 or > 60 years; n = 125), and age at first birth (< 12 or > 50 years; n = 6). Our final study sample comprised 160,881 participants who completed a baseline questionnaire between 1991 and 2008. A flowchart of the study sample is presented in Supplementary Fig. 1.

For breast cancer survival analyses, we included 7,832 women diagnosed with incident postmenopausal breast cancer between 1991 and 2020, excluding those without breast cancer and 12 who were diagnosed post-mortem or after emigration.

### Exposure and covariates

Information on MHT use, including ever use, current use, age at first use, and duration of use, was obtained from questionnaires. Furthermore, MHT was categorized into specific MHT regimens, with participants providing this information via timeline tables and a photo booklet of all available Norwegian MHT brands. We then categorized MHT use into EPT and ET and calculated cumulative estradiol (E2)- and norethisterone (NETA)-equivalent doses. Patients who previously used EPT were excluded from the ET users’ group, leaving a category of patients who had only used unopposed estrogen. MHT status (ever/never, current/former/never) and duration were updated from the follow-up questionnaires to the last non-missing values at start of follow-up.

Covariates of interest were extracted from the questionnaires, and the last non-missing value before inclusion was used. We selected covariates of interest a priori and used directed acyclic graphs (DAGs) to depict their assumed causal relationship with exposure and outcome, thereby identifying potential confounding factors adjusted for in the multivariable models [43]. These covariates included age (used as time metric), body mass index (BMI; continuous), parity (0, 1, 2, ≥ 3) and age at first birth (< 25, 25–29, ≥ 30 years; combined into one variable), age at menarche (continuous), family history of breast cancer (none, mother and sister, only mother, only sister), physical activity (low, moderate, high), smoking status (current, former, never), and education (< 9, 10–12, 13–16, ≥ 17 years of schooling). Separate DAGs were performed for three outcome variables: overall breast cancer incidence, mortality, and survival (Supplementary Figs.2, 3, 4, respectively). To facilitate comparisons with previous literature, supplementary analyses on breast cancer survival were carried out, whereby models were adjusted for tumor stage (I, II, III, IV), surgical status (lumpectomy, mastectomy, other), and age at diagnosis (Model 1), as well as adding these variables to the main multivariable-adjusted analyses (Model 2; Supplementary Table7).

### Outcome

Incident breast cancer cases were identified through passive linkage to the Cancer Registry of Norway and classified according to the International Classification of Diseases 10th revision (ICD-10, C50). Breast cancer-specific deaths were identified through the Cause of Death Registry, and emigration status was supplemented by the Central Population Register. These registries provide annual endpoint information, including the date of cancer diagnosis, death, emigration, and cause of death.

Information on tumor markers, characteristics, and mammography screening was obtained from the Cancer Registry of Norway. The registry routinely extracts information on ER and PR status from pathology reports. Receptor status was assessed using IHC by nationwide pathological departments. Before January 2012, ER-negative tumors were defined using a threshold of < 10% reactivity. Owing to alterations in the national treatment guidelines since February 2012, the threshold shifted to < 1% reactivity. This study employed these cutoff points. HER2 status was ascertained using IHC and/or in situ hybridization (ISH) techniques. Tumors exhibiting no or weak immunostaining were classified as HER2-negative, while those exhibiting moderate or strong immunostaining were classified as HER2-positive. ISH was used to verify cases of moderate immunostaining. Finally, molecular subtypes were approximated using the IHC surrogate definition from the St. Gallen 2013 Expert Panel: luminal A-like (ER + PR + HER2-), luminal B-like (ER + PR + HER2- or ER + PR- HER2 + or ER + PR + HER2 +), HER2-enriched (ER- PR- HER2 +), and triple-negative (ER- PR- HER2-) [6]. The Cancer Registry of Norway is estimated to be 98.8% complete [44].

### Menopausal status

Participants were considered postmenopausal if their menstrual period had stopped naturally or surgically by bilateral oopherectomy. Those with unknown menopausal age, who reported irregular menses, hysterectomy, or MHT use, were considered postmenopausal at age 53. This cutoff was used to maintain consistency with the Million Women Study convention [7], and previous NOWAC publications [45, 46]. For current smokers, this age was adjusted to 51 years, as smoking can reduce the menopausal age by approximately 2 years [47].

### Follow-up

For incidence and mortality analyses, follow-up began at the date of the baseline questionnaire for postmenopausal participants. If menopause occurred later, follow-up began at the age of menopause, age at MHT initiation, or age 53 [51 for smokers]. MHT use at study entry refers to the last questionnaire completed before inclusion in the regression analysis. Exit time was defined as the date of cancer diagnosis, death, emigration, or end of follow-up, whichever occurred first. For breast cancer survival analyses, follow-up was from diagnosis until death, emigration, or end of follow-up. Participants were censored at 10 years post-diagnosis to retrieve the 10-year risk of death among patients with breast cancer as a measure of survival. The cause and date of death were updated until April 30, 2022, and breast cancer incidence updated until December 31, 2020.

### Statistical analyses

Cox proportional hazard regression models were used to estimate hazard ratios (HRs) and 95% confidence intervals (CIs) for associations between MHT use and the incidence, mortality, and survival of overall and intrinsic-like breast cancer subtypes, using age as the underlying time scale. Distinct regression models were fitted for each subtype outcome, censoring patients diagnosed with or dying from a different subtype [48]. The Cox proportional hazard’s assumption was evaluated by graphical inspection of Schoenfeld residuals and survival time [49]. To account for variations in cumulative estrogen and progestin doses due to age differences, regression models included age at enrollment as a stratum variable.

A total of 22,434 (14%) participants had missing information on at least one covariate. The percentages of missing covariates are listed in Table 1. Assuming these variables were missing at random, we performed multiple imputation by chained equations (MICE) to handle the missing data. A MICE model was executed for each subtype outcome (overall breast cancer and intrinsic-like subtypes) within the incidence, mortality, and survival analytical samples. MICE models included all covariates, a MHT variable (never, current, or former use of ETP, ET, or an unknown type), age at study entry, a binary outcome variable, and the Nelson–Aalen cumulative hazard estimator. MICE models were constructed using predictive mean matching for continuous variables (BMI, age at menarche, and age at first birth), ordered logistic regression for ordinal categorical variables (physical activity and education), and multinomial logistic regression for non-ordinal categorical variables (smoking status). Family history of breast cancer and parity were used as auxillary variables. To reduce sampling variability during the imputation process, 20 duplicate datasets were created [50]. The estimates and standard errors in the imputed datasets were combined using Rubin´s rule to account for within- and between-imputation variances [51]. Age-adjusted and complete-case analyses were performed as sensitivity analyses.

**Table 1:** Descriptives of study sample according to MHT use at study entry

	MHT use at study entry			
	Never MHT	Ever EPT use	Ever ET use only^1^	Ever unknown type
	Mean ± SD or n (%)			
Number of women, n (%)	102,058 (63.4)	40,778 (25.4)	5,630 (3.5)	12,415 (7.7)
Invasive breast cancer cases	4,297 (4.1)	2,599 (6.4)	262 (4.7)	686 (5.5)
Age at study entry (yrs)	53.9 ± 0.01	53.2 ± 0.03	53.4 ± 0.07	52.6 ± 0.06
Age at menarche (yrs)	13.3 ± 0.00	13.3 ± 0.01	13.2 ± 0.02	13.3 ± 0.01
Missing, n (%)	1,797 (1.8)	524 (1.3)	86 (1.5)	259 (2.1)
Age at menopause (yrs)	49.5 ± 0.02	49.7 ± 0.03	46.2 ± 0.08	48.3 ± 0.06
Missing, n (%)	47,676 (46.7)	10,731 (26.3)	1,193 (21.2)	4,075 (32.8)
Age at first birth (yrs)^2^	24.2 ± 0.02	23.8 ± 0.02	23.3 ± 0.06	23.4 ± 0.04
Missing, n (%)	49 (0.1)	2 (0.0)	0 (0.0)	0 (0.0)
Parity	2.3 ± 0.00	2.2 ± 0.01	2.1 ± 0.01	2.3 ± 0.01
Missing, n (%)	0 (0)	0 (0)	0 (0)	0 (0)
BMI (kg/m^2^)	24.7 ± 0.01	24.3 ± 0.02	24.7 ± 0.05	24.6 ± 0.04
Missing, n (%)	2,167 (2.1)	706 (1.7)	119 (2.1)	365 (2.9)
Alcohol consumption (g/day)	3.49 ± 0.02	4.23 ± 0.03	4.03 ± 0.07	3.46 ± 0.05
Missing, n (%)	4,088 (4.0)	2,133 (5.2)	282 (5.0)	845 (6.8)
Education, n (%)				
≤ 9 yrs	22,535 (22.1)	7,793 (19.1)	1,108 (19.7)	3,584 (28.9)
10–12 yrs	32,513 (31.9)	13,593 (33.3)	1,946 (34.6)	3,967 (32.0)
13–16 yrs	26,796 (26.3)	11,070 (27.2)	1,472 (26.2)	2,586 (20.8)
≥ 17 yrs	14,407 (14.1)	6,148 (15.1)	774 (13.8)	1,264 (10.2)
Missing	5,807 (5.7)	2,174 (5.3)	330 (5.9)	1,014 (8.2)
Family history of breast cancer, n (%)				
None	94,481 (92.6)	37,774 (92.6)	5,198 (92.3)	11,491 (92.6)
Mother and sister	301 (0.3)	108 (0.3)	10 (0.2)	40 (0.3)
Mother	5,176 (5.1)	1,996 (4.9)	301 (5.4)	573 (4.6)
Sister	2,100 (2.1)	900 (2.2)	121 (2.2)	311 (2.5)
Missing	0 (0)	0 (0)	0 (0)	0 (0)
Smoking status, n (%)				
Never	37,843 (37.1)	12,842 (31.5)	1,870 (33.2)	3,964 (31.9)
Former	33,783 (33.1)	15,368 (37.7)	2,118 (37.6)	4,099 (33.0)
Current	29,502 (28.9)	12,358 (30.3)	1,602 (28.5)	4,109 (33.1)
Missing	930 (0.9)	210 (0.5)	40 (0.7)	243 (2.0)
Physical activity, n (%)				
Low	22,411 (22.0)	9,386 (23.0)	1,382 (24.6)	3,026 (24.4)
Moderate	54,820 (53.7)	22,735 (55.8)	3,053 (54.2)	6,001 (48.3)
High	17,013 (16.7)	6,507 (16.0)	885 (15.7)	1,782 (14.4)
Missing	7,814 (7.7)	2,150 (5.3)	310 (5.5)	1,606 (12.9)
Oral contraceptive use, n (%)				
Never	43,708 (42.8)	16,551 (40.6)	2,496 (44.3)	5,466 (44.0)
Ever	54,967 (53.9)	23,584 (57.8)	3,015 (53.6)	6,406 (51.6)
Missing	3,383 (3.3)	643 (1.6)	119 (2.1)	543 (4.4)

^1^Never EPT users

^2^Among parous women

*EPT* estrogen-progestin therapy*, ET* estrogen therapy, *MHT* menopausal hormone therapy*, BMI* body mass index

All *p*-values were two-sided with a type I error rate of 5%. Heterogeneity across breast cancer subtypes was tested using the Wald test after a duplication method for competing risk analysis [52, 53]. All statistical analyses were performed using STATA version 17.0 (StataCorp, College Station, TX, USA).

## Results

A total of 160,881 participants were followed for a median of 15.8 years for breast cancer incidence and 18.0 years for breast cancer-specific mortality. At study entry (in median year 2004), these participants were free from breast cancer and were postmenopausal. Among them, 40,974 (26%) were current MHT users (29,522 EPT and 4,370 ET), 17,849 (11%) were former users (11,256 EPT and 1,260 ET), and 102,058 (63%) had never used MHT at study entry. For the 10-year breast cancer-specific survival estimates, 7,832 patients with incident breast cancer (diagnosed in median year 2012) were followed for a median of 8.5 years. Descriptive statistics for the study sample are presented in Table 1, with case characteristics in Supplementary Tables 1 and 2. Notably, MHT users had higher alcohol consumption, higher education, were less likely to smoke, and were more likely to have used oral contraceptives than non-users.

### Breast *cancer* incidence

Ever and current use of MHT and EPT at study entry were associated with increased risk of overall, luminal A-like, and luminal B-like breast cancer compared with never use (Table 2), with associations varying by subtype (*p*_heterogeneity_ = 0.02 and 0.04 for current MHT and EPT use, respectively). The highest HR was for the luminal A-like subtype (HR 1.41; 95% CI 1.31–1.52 for current EPT use). A significant trend for duration of use was observed for the overall, luminal A-like, and luminal B-like subtypes, with HRs increasing by 4%, 4%, and 2% per year of EPT use, respectively. Former EPT and ET use was associated with decreased risk of luminal A-like (HR 0.86; 95% CI 0.75–0.99) and overall breast cancer (HR 0.68; 95% CI 0.49–0.94) compared with never use. Increasing associations with overall, luminal A-like, and luminal B-like breast cancer were observed with increasing cumulative estrogen doses. Cumulative progestin dose was associated with overall (HR 1.66; 95% CI 1.52–1.82), luminal A-like (HR 1.87; 95% CI 1.65–2.12), luminal B-like (HR 1.60; 95% CI 1.30–1.97), and HER2-enriched (HR 1.79; 95% CI 1.08–2.98 for > 2 g NETA equivalence) breast cancer. High estrogen dose (≥ 5 g) combined with low progestin dose (< 1 g) was associated with a twofold increased risk of TNBC (HR 2.23; 95% CI 1.22–4.09). Supplementary Tables 3 and 4 provide corresponding results for non-imputed, age-adjusted and multivariable-adjusted complete-case analyses.

**Table 2:** MHT use at study entry and breast cancer incidence by intrinsic-like subtypes

	Breast cancer overall (n = 7,844)		Luminal A-like (n = 3,784)		Luminal B-like (n = 1,480)		HER2 + (n = 264)		TNBC (n = 500)		*p*_het_^2^
	n cases	HR (95% CI)^1^	n cases	HR (95% CI)^1^	n cases	HR (95% CI)^1^	n cases	HR (95% CI)^1^	n cases	HR (95% CI)^1^	
**MHT use overall**											
Never use	4,297	Ref	2,113	Ref	845	Ref	155	Ref	297	Ref	
Ever use	3,547	1.24 (1.18–1.29)	1,671	1.16 (1.10–1.25)	635	1.13 (1.02–1.26)	109	1.07 (0.83–1.37)	203	1.03 (0.86–1.23)	0.51
Current	2,782	1.35 (1.29–1.42)	1,310	1.32 (1.23–1.41)	464	1.17 (1.04–1.31)	77	1.05 (0.80–1.39)	139	0.99 (0.80–1.21)	0.02
Former	765	0.95 (0.88–1.02)	361	0.83 (0.75–0.93)	171	1.04 (0.88–1.23)	32	1.12 (0.76–1.64)	64	1.12 (0.85–1.48)	0.08
Duration											
< 5 yrs	2,250	1.16 (0.10–1.22)	984	1.06 (0.98–1.14)	416	1.13 (1.00–1.27)	78	1.14 (0.86–1.50)	128	0.97 (0.79–1.20)	
≥ 5 yrs	1,243	1.40 (1.31–1.49)	656	1.37 (1.26–1.50)	212	1.15 (0.99–1.34)	30	0.94 (0.63–1.40)	71	1.13 (0.87–1.46)	
Per 1 yr	7,790	1.03 (1.03–1.04)	3,753	1.03 (1.02–1.04)	1,473	1.02 (1.00–1.03)	263	1.00 (0.96–1.04)	496	1.01 (0.98–1.03)	0.04
**EPT use**											
Never use	4,297	Ref	2,113	Ref	845	Ref	155	Ref	297	Ref	
Ever use	2,599	1.32 (1.25–1.38)	1,248	1.26 (1.17–1.35)	464	1.19 (1.06–1.34)	82	1.16 (0.88–1.52)	147	1.08 (0.88–1.32)	0.45
Current	2,120	1.44 (1.36–1.52)	1,012	1.41 (1.31–1.52)	352	1.23 (1.09–1.40)	58	1.09 (0.81–1.49)	107	1.06 (0.85–1.32)	0.04
Former	479	0.96 (0.87–1.05)	236	0.86 (0.75–0.99)	112	1.09 (0.89–1.33)	24	1.34 (0.86–2.06)	40	1.13 (0.81–1.57)	0.11
Duration											
< 5 yrs	1,559	1.22 (1.15–1.29)	688	1.11 (1.02–1.22)	288	1.18 (1.03–1.35)	56	1.22 (0.89–1.66)	91	1.04 (0.82–1.32)	
≥ 5 yrs	1,028	1.49 (1.39–1.60)	553	1.48 (1.35–1.63)	175	1.22 (1.03–1.44)	25	1.01 (0.66–1.54)	55	1.12 (0.84–1.50)	
Per 1 yr	6,884	1.04 (1.03–1.05)	3,354	1.04 (1.03–1.05)	1,308	1.02 (1.01–1.04)	236	1.01 (0.97–1.06)	443	1.01 (0.98–1.04)	0.05
**ET use only**											
Never use	4,297	Ref	2,113	Ref	845	Ref	155	Ref	297	Ref	
Ever use	262	0.96 (0.85–1.09)	122	0.89 (0.74–1.06)	52	0.97 (0.73–1.29)	12	1.25 (0.69–2.26)	15	0.80 (0.48–1.35)	0.68
Current	224	1.04 (0.91–1.19)	102	0.95 (0.78–1.16)	47	1.12 (0.83–1.50)	9	1.18 (0.60–2.32)	12	0.81 (0.45–1.45)	0.68
Former	38	0.68 (0.49–0.94)	20	0.65 (0.42–1.01)	5	0.43 (0.18–1.04)	3	1.51 (0.48–4.74)	3	0.76 (0.24–2.37)	0.39
Duration											
< 5 yrs	164	0.97 (0.83–1.13)	78	0.95 (0.76–1.20)	29	0.90 (0.62–1.30)	8	1.34 (0.66–2.72)	8	0.70 (0.35–1.41)	
≥ 5 yrs	96	0.95 (0.78–1.17)	43	0.78 (0.58–1.06)	22	1.05 (0.69–1.61)	4	1.13 (0.42–3.05)	7	0.98 (0.46–2.08)	
Per 1 yr	4,557	0.99 (0.97–1.01)	2,234	0.98 (0.95–1.01)	896	1.00 (0.96–1.04)	167	0.99 (0.89–1.10)	312	0.98 (0.90–1.06)	0.85
**Cumulative dose**											
Never use	4,297	Ref	2,113	Ref	845	Ref	155	Ref	297	Ref	
Estrogen (E2-equivalence)											
< 5 g	1,999	1.21 (1.15–1.28)	948	1.23 (1.14–1.33)	347	1.13 (1.00–1.29)	69	1.29 (0.97–1.74)	112	1.01 (0.81–1.27)	0.49
5**–**10 g	827	1.36 (1.26–1.47)	399	1.45 (1.29–1.62)	154	1.39 (1.16–1.66)	22	1.24 (0.78–1.97)	48	1.21 (0.88–1.66)	0.66
> 10 g	192	1.51 (1.30–1.75)	103	1.79 (1.46–2.18)	34	1.46 (1.03–2.07)	5	1.39 (0.57–3.43)	6	0.74 (0.33–1.66)	0.19
Progestin (NETA-equivalence)											
< 1 g	1,411	1.20 (1.13–1.28)	634	1.16 (1.06–1.27)	257	1.18 (1.02–1.36)	46	1.19 (0.85–1.66)	92	1.18 (0.93–1.50)	0.97
1**–**2 g	695	1.36 (1.25–1.47)	361	1.55 (1.38–1.74)	112	1.19 (0.97–1.45)	18	1.18 (0.72–1.95)	33	0.99 (0.68–1.43)	0.04
> 2 g	608	1.66 (1.52–1.82)	304	1.87 (1.65–2.12)	107	1.60 (1.30–1.97)	18	1.79 (1.08–2.98)	24	1.02 (0.66–1.56)	0.09
E2 dose < 5 g											
NETA dose < 1 g	1,306	1.20 (1.14–1.28)	589	1.16 (1.06–1.27)	237	1.17 (1.01–1.35)	43	1.20 (0.85–1.69)	79	1.09 (0.85–1.41)	0.98
NETA dose ≥ 1 g	439	1.47 (1.33–1.63)	233	1.73 (1.51–1.98)	66	1.20 (0.93–1.55)	12	1.36 (0.75–2.48)	18	0.93 (0.57–1.50)	0.03
E2 dose ≥ 5 g											
NETA dose < 1 g	93	1.20 (0.98–1.48)	40	1.14 (0.84–1.57)	18	1.29 (0.80–2.05)	3	1.30 (0.41–4.09)	11	2.23 (1.22–4.09)	0.27
NETA dose ≥ 1 g	862	1.49 (1.38–1.61)	431	1.66 (1.48–1.84)	153	1.44 (1.20–1.72)	23	1.39 (0.88–2.19)	39	1.04 (0.74–1.48)	0.09

^1^Adjusted for age (underlying time scale), BMI, parity, age at first birth, age at menarche, family history, smoking, physical activity, education

^2^*p* heterogeneity between intrinsic-like subtypes; Wald test by competing risks analysis

*CI* confidence interval*, ET,* estrogen therapy*, EPT* estrogen-progestin therapy*, E2* estradiol*, HER2* human epidermal growth factor receptor 2*, HR* hazard ratio *MHT* menopausal hormone therapy *NETA* norethisterone acetate*, TNBC* triple-negative breast cancer

### Breast *cancer* mortality

Among the entire study sample, MHT use at study entry increased risk of overall breast cancer-specific mortality compared to never use (Table 3; HR 1.61; 95% CI 1.36–1.91 among current EPT users). Ever (HR 1.74; 95% CI 1.24–2.44) and current use (HR 2.15; 95% CI 1.51–3.05) of EPT at study entry were associated with increased risk of dying from luminal A-like breast cancer.

**Table 3:** MHT use at study entry and breast cancer-specific mortality by intrinsic-like subtypes

	Breast cancer deaths overall (n = 721)		Luminal A-like (n = 163)		Luminal B-like (n = 113)		HER2-enriched (n = 33)		TNBC (n = 81)		*p*_het_^2^
	n	HR (95% CI)^1^	n	HR (95% CI)^1^	n	HR (95% CI)^1^	n	HR (95% CI)^1^	n	HR (95% CI)^1^	
**MHT use overall**											
Never use	392	Ref	82	Ref	64	Ref	20	Ref	54	Ref	
Ever use	329	1.27 (1.09–1.47)	81	1.52 (1.11–2.07)	49	1.11 (0.76–1.61)	13	1.00 (0.49–2.04)	27	0.72 (0.45–1.15)	0.10
Current	268	1.48 (1.26–1.73)	65	1.82 (1.31–2.54)	39	1.29 (0.86–1.94)	13	1.43 (0.70–2.94)	16	0.60 (0.34–1.06)	0.03
Former	61	0.78 (0.60–1.03)	16	0.91 (0.53–1.56)	10	0.71 (0.36–1.39)	0	-	11	1.01 (0.52–1.94)	0.80
Duration											
< 5 yrs	220	1.29 (1.09–1.53)	43	1.28 (0.88–1.86)	31	1.10 (0.71–1.69)	10	1.15 (0.53–2.50)	16	0.65 (0.37–1.15)	
≥ 5 yrs	104	1.22 (0.98–1.52)	35	1.86 (1.24–2.78)	17	1.08 (0.63–1.86)	3	0.73 (0.11–1.49)	11	0.89 (0.46–1.72)	
Per 1 yr	716	1.02 (1.00–1.04)	160	1.06 (1.02–1.09)	112	1.01 (0.96–1.06)	33	0.95 (0.83–1.09)	81	0.98 (0.92–1.06)	0.13
**ETP use**											
Never use	392	Ref	82	Ref	64	Ref	20	Ref	54	Ref	
Ever use	237	1.35 (1.14–1.59)	62	1.74 (1.24–2.44)	37	1.23 (0.81–1.86)	11	1.25 (0.59–2.64)	20	0.78 (0.46–1.31)	0.13
Current	208	1.61 (1.36–1.91)	54	2.15 (1.51–3.05)	31	1.44 (0.93–2.22)	11	1.70 (0.80–3.62)	14	0.74 (0.41–1.33)	0.05
Former	29	0.62 (0.43–0.91)	8	0.77 (0.37–1.60)	6	0.71 (0.30–1.64)	0	-	6	0.90 (0.38–2.11)	0.94
Duration											
< 5 yrs	152	1.38 (1.14–1.67)	30	1.42 (0.93–2.18)	23	1.27 (0.78–2.05)	9	1.60 (0.72–3.59)	11	0.69 (0.36–1.32)	
≥ 5 yrs	84	1.28 (1.01–1.62)	31	2.16 (1.42–3.29)	14	1.15 (0.64–2.08)	2	0.63 (0.15–2.73)	9	0.94 (0.46–1.92)	
Per 1 yr	628	1.02 (1.00–1.05)	143	1.07 (1.04–1.11)	101	1.02 (0.97–1.08)	31	0.95 (0.82–1.10)	74	0.99 (0.92–1.07)	0.10

^1^Adjusted for age (underlying time scale), BMI, parity, age at first birth, age at menarche, family history, smoking, physical activity, education

^2^*p* heterogeneity between intrinsic-like subtypes; Wald test by competing risks analysis

*CI* confidence interval, *ETP* estrogen-progestin therapy, *HER2* human epidermal growth factor receptor 2, *HR* hazard ratio, *MHT* menopausal hormone therapy, *TNBC* triple-negative breast cancer

The association with breast cancer mortality increased by 2% per year of EPT use, and ≥ 5 years of EPT use was associated with a twofold risk of dying from luminal A-like breast cancer (HR 2.16; 95% CI 1.42–3.29). No association was observed between MHT use and luminal B-like, HER2-enriched, or TNBC mortality. Associations between current MHT use and breast cancer mortality varied across intrinsic-like subtypes (*p*_heterogeneity_ = 0.03). Complete-case analysis results are presented in Supplementary Table 5.

### Breast *cancer* survival

Among patients with breast cancer, MHT use was associated with increased risk of death from luminal A-like cancer, albeit statistically non-significantly, thus lower 10-year survival compared with non-users (Table 4; HR death 1.36; 95% CI 0.94–1.99 for current EPT use at study entry).

**Table 4:** MHT use at study entry and 10-year survival by intrinsic-like subtypes

	Breast cancer deaths overall (n = 634)		Luminal A-like (n = 148)		Luminal B-like (n = 104)		HER2 + (n = 32)		TNBC (n = 81)		*p*_het_^3^
	n	HR (95% CI)^1,2^	n	HR (95% CI)^1,2^	n	HR (95% CI)^1,2^	n	HR (95% CI)^1,2^	n	HR (95% CI)^1,2^	
**MHT use overall**											
Never use	356	Ref	76	Ref	62	Ref	19	Ref	54	Ref	
Ever use	278	0.95 (0.81–1.11)	72	1.20 (0.86–1.67)	42	0.78 (0.52–1.17)	13	0.90 (0.44–1.86)	27	0.56 (0.35–0.90)	0.10
Current	226	0.97 (0.82–1.15)	58	1.28 (0.90–1.82)	32	0.77 (0.50–1.19)	13	1.14 (0.55–2.35)	16	0.41 (0.24–0.73)	0.02
Former	52	0.85 (0.63–1.13)	14	0.95 (0.53–1.68)	10	0.82 (0.42–1.62)	0	-	11	1.13 (0.59–2.20)	0.78
Duration											
< 5 yrs	181	0.95 (0.79–1.14)	38	1.04 (0.70–1.54)	26	0.78 (0.49–1.25)	10	1.05 (0.48–2.29)	16	0.52 (0.30–0.92)	
≥ 5 yrs	93	0.94 (0.74–1.18)	32	1.43 (0.93–2.19)	15	0.74 (0.42–1.32)	3	0.65 (0.19–2.24)	11	0.66 (0.34–1.28)	
Per 1 yr	630	0.99 (0.97–1.02)	146	1.03 (0.99–1.07)	103	0.98 (0.92–1.04)	32	0.93 (0.81–1.07)	81	0.95 (0.88–1.03)	0.12
**ETP use**											
Never use	356	Ref	76	Ref	62	Ref	19	Ref	54	Ref	
Ever use	201	0.94 (0.79–1.12)	54	1.24 (0.86–1.77)	31	0.78 (0.50–1.22)	11	1.06 (0.49–2.26)	20	0.57 (0.34–0.96)	0.14
Current	175	0.99 (0.82–1.19)	47	1.36 (0.94–1.99)	25	0.78 (0.49–1.26)	11	1.27 (0.59–2.72)	14	0.48 (0.26–0.87)	0.05
Former	26	0.70 (0.47–1.05)	7	0.77 (0.35–1.67)	6	0.79 (0.34–1.85)	0	-	6	1.03 (0.44–2.44)	0.92
Duration											
< 5 yrs	126	0.97 (0.79–1.19)	25	1.00 (0.63–1.58)	19	0.83 (0.49–1.40)	9	1.38 (0.61–3.09)	11	0.52 (0.27–1.01)	
≥ 5 yrs	74	0.90 (0.70–1.16)	28	1.53 (0.98–2.39)	12	0.71 (0.38–1.33)	2	0.52 (0.12–2.27)	9	0.65 (0.32–1.34)	
Per 1 yr	556	0.99 (0.96–1.02)	129	1.04 (1.00–1.09)	93	0.98 (0.92–1.04)	30	0.93 (0.79–1.08)	74	0.95 (0.88–1.03)	0.08

^1^HRs of breast-cancer specific death

^2^Adjusted for age (underlying time scale), BMI, parity, age at first birth, age at menarche, family history, smoking, physical activity, education

^3^*p* heterogeneity between intrinsic-like subtypes; Wald test by competing risks analysis

*CI* confidence interval, *ETP* estrogen-progestin therapy, *HER2* human epidermal growth factor receptor 2, *HR* hazard ratio, *MHT* menopausal hormone therapy, *TNBC* triple-negative breast cancer

Similarly, the duration of EPT use at study entry was associated with an increased risk of death from luminal A-like breast cancer (HR death 1.04; 95% CI 1.00–1.09 per year increment). Ever (HR death 0.57; 95% CI 0.34–0.96) and current use (HR death 0.48; 95% CI 0.26–0.87) of EPT at study entry was associated with decreased risk of death from TNBC compared with never users. Moreover, current MHT use was differentially associated with survival across intrinsic-like subtypes (*p*_heterogeneity_ = 0.02). Complete-case analysis findings are presented in Supplementary Table 6. Adjustment for tumor stage, surgical status and age at diagnosis did not substantially alter risk estimates (Supplementary Table 7).

## Discussion

In this prospective cohort study with 160,881 participants, 7,844 incident breast cancer cases, and 721 breast cancer-specific deaths, MHT use was associated with increased risks of incident and fatal overall and luminal A-like breast cancers. Longer duration of use and higher cumulative doses of estrogen and progestin at study entry were associated with higher risks of overall, luminal A-like, and luminal B-like breast cancers, indicating a dose–response relationship. We observed differences in risk based on recency, where the strongest HRs were observed with current use at study entry. Despite positive associations between MHT use and breast cancer incidence and mortality, we did not observe worse survival among patients with breast cancer who were pre-diagnostic MHT users. Although based on small numbers, there were indications that MHT use at study entry was associated with a decreased risk of breast cancer-specific death among patients with TNBC. This study provides insights into the nuanced effects of MHT on etiology and progression of breast cancer subtypes.

Our findings on breast cancer incidence align with the empirically grounded consensus that MHT use increases breast cancer risk [13, 21], with effect estimates among current users similar to those of large, prospective studies [9, 12, 21]. Consistent with previous reports, past use was not associated with increased risk of incident or fatal disease [7]. Moreover, the association with an increased risk of luminal subtypes is also reflected in previous studies [16, 17, 19, 21]. We did not observe any association between general MHT use and HER2-enriched or TNBC subtypes, consistent with several studies [16, 17, 19]. However, we observed an association between high cumulative estrogen combined with low cumulative progestin dose and incident TNBC, and increasing cumulative progestin dose and incident HER2-enriched breast cancer. These results are based on small numbers and should be interpreted cautiously. Our results predominantly did not suggest any associations with ET use.

The findings on overall breast cancer mortality and survival partly reflect those reported in existing literature. Our results align with reports that MHT is associated with an increased risk of death from breast cancer among the entire study population [23, 25, 26]. In contrast, and in agreement with previous publications, pre-diagnostic MHT use at study entry was not associated with an increased risk of breast cancer-specific death among patients with breast cancer. There were some indication of inverse associations, as previous studies have disclosed [24, 29–33, 35], but the results were statistically non-significant. Contrary to these publications, the absence of statistically significant inverse associations with overall breast cancer survival in the present study may be attributed to different recruitment periods. Due to a shift toward increased use of low-dose EPT formulations and non-oral MHT regimens in the early 2000s [54, 55], one could expect studies with recruitment after the millennium shift to report risk estimates of different magnitude than those of older age. In our study with start of follow-up in median year 2004, we anticipate a mixture of user patterns seen prior to and following the millennium shift. A recent publication with contemporary MHT formulations have reported increased risk of comparable magnitude to those of older studies [21]. However, studies evaluating MHT use and breast cancer-specific mortality and survival are generally from earlier recruitment periods and the associations between newer MHT formulations and these outcomes are not well known.

Controlling for mammography screening in analyses of breast cancer survival and mortality has been advocated [25, 26], as MHT users undergo mammography more frequently than non-users [56, 57] and screen-detected cancers tend to be of more favorable grade, early stage, and hormone receptor-positive [56, 58, 59]. The increased survival observed in previous studies could be attributed to mammography screening, producing lead-time bias due to early detection and length bias owing to the identification of slow-growing tumors. However, increased survival has been reported in studies both controlling for mammography [31–33] and those that did not [24, 29]. Furthermore, it has been argued that increased survival associated with MHT use is not explained by mammographic surveillance but by biological mechanisms [33]. We chose not to adjust for mammographic screening in our analysis, as we do not consider it a confounder, but rather a possible intermediate variable in the causal pathway between MHT use and breast cancer subtypes. However, differences in health-seeking behaviors and screening attendance could be related to socioeconomic status [60], affecting MHT use [61] and survival rates. Therefore, we adjusted for educational level. Unfortunately, education level was the only available indicator to capture socioeconomic status and its impact on MHT use and breast cancer death. Thus, residual confounding cannot be excluded. Moreover, unmeasured confounding arising from non-exchangeability between MHT users and non-users, i.e. differences in MHT users and non-users that affect the outcome, cannot be definitively ruled out.

In accordance with mammographic screening, we did not adjust for clinical characteristics such as stage or treatment in our main analyses, as these factors are intermediates between MHT use and breast cancer survival. Evidence supporting a biological chronology in which the molecular subtype precedes tumor characteristics is found in studies where intrinsic-like subtypes have been assessed in pre-cancerous lesions [62, 63]. Upon adjusting for stage, surgical status and age at diagnosis in a supplementary analysis, effect estimates were substantially unaltered, underscoring that the observed associations were not explained by such clinical characteristics.

Another explanation for the opposing risk estimates on overall breast cancer mortality and survival could be the presence of collider stratification bias, also referred to as index event bias, which is introduced when conditioning on an intermediate variable between the exposure and outcome, coupled with unmeasured confounding factors affecting the mediator’s impact on the outcome [64–67]. In our scenario, a cancer or subtype-specific cancer diagnosis is an intermediate variable between MHT use and breast cancer survival, and genetic susceptibility to breast cancer represents unmeasured confounding for the effect of a subtype diagnosis on death from breast cancer [68, 69]. We considered this by adjusting for family history of breast cancer, a surrogate variable for genetic susceptibility. However, we cannot completely rule out residual confounding and selection bias. Hence, these results must be interpreted without drawing causal conclusions.

Our findings indicated a reduced HR of death among patients with TNBC who were MHT users pre-diagnosis. The BCAC pooled analysis also demonstrated increased survival among patients with TNBC, with a HR of 0.64 (95% CI 0.48–0.85) of death from TNBC among current EPT users [29]. However, in contrast to our study, they revealed similar effect estimates for all subtypes and did not detect heterogeneity by intrinsic-like subtypes. One study demonstrated an increased risk of incident TNBC with current MHT use [21], aligning with our finding of an association between high cumulative estrogen combined with low cumulative progestin intake and incident TNBC. Potential biological mechanisms linking estrogen and progestin to TNBC as alternatives to the classical ER/PR pathway include receptor conversion, alternative estrogen-binding receptors, androgen receptor stimulation, and paracrine pathways [70]. Although several possible mechanisms exist whereby MHT use could exert associations in triple-negative tumor initiation and progression, the direction of these effects remain unclear.

Our study has some limitations. First, we were limited by small subsamples, particularly in the analyses of mortality and survival of the less common receptor-negative subtypes. This was partly due to missing data on receptor status and the small number of breast cancer-specific deaths. We chose not to perform multiple imputations on receptor status because imputing outcome data is a subject of controversy [71]. The limited statistical power in these analyses precludes causal interpretations. Second, we used self-reported information on MHT use and covariates. Although a potential for misclassification exists, a validation study on MHT use in the NOWAC cohort demonstrated valid information on current MHT use at baseline and menopausal status among women aged 48–62 [46]. Third, multiple imputations were performed on missing covariate data under the assumption that these variables were missing at random. Similar effect estimates in sensitivity analyses on complete-case data support the robustness of our assumptions; however, we cannot rule out the possibility that some information was missing not at random; thus, our estimates may not be free from bias. Fourth, a multi-state survival model could be a viable approach in understanding the biology behind pre-diagnostic MHT use and breast cancer progression [72]. However, due to the multiple outcomes among breast cancer subtypes, employing this model was outside the scope of our study. Lastly, as we only had information on the first incident breast cancer subtype, some deaths could have resulted from converted or recurrent subtypes that differed from those identified at the initial diagnosis.

## Conclusions

We have demonstrated that MHT use was associated with a small increased risk of incident and fatal overall and luminal breast cancers. However, the relationship between MHT use and breast cancer survival is complex. While pre-diagnostic MHT use was not associated with overall breast cancer survival, it was associated with increased survival among patients with TNBC. These findings underscore the intricate relationship between MHT and breast cancer outcomes across subtypes. Further research is needed to elucidate the mechanisms behind differential effects on breast cancer mortality and survival associated with MHT use.

## Supplementary Information

Additional file 1

## Data Availability

No datasets were generated or analysed during the current study.

## Abbreviations

CI: Confidence interval
ER: Estrogen receptor
ET: Estrogen therapy
EPT: Estrogen-progestin therapy
HR: Hazard ratio
HER2: Human epidermal growth factor receptor 2
IHC: Immunohistochemistry
ISH: In situ Hybridization
MHT: Menopausal hormone therapy
MICE: Multiple imputation by chained equations
NETA: Norethisterone acetate
NOWAC: The Norwegian Women and Cancer Study
PR: Progesterone receptor
TNBC: Triple-negative breast cancer
