# Supplementary material for: Menopausal hormone therapy and incidence, mortality, and survival of breast cancer subtypes: a prospective cohort study
Source: Breast Cancer Res. 2024 Nov 4;26:151. doi: 10.1186/s13058-024-01897-4 (PMC11536865; doi:10.1186/s13058-024-01897-4)
Supplement: Supplementary file 1 — Additional file 1 [file 13058_2024_1897_MOESM1_ESM.docx]

# Supplementary tables

**Supplementary Table 1**. Clinical descriptives of cases

|  | **Overall breast cancer cases** | **Luminal A-like cases** | **Luminal B-like cases** | **HER2+ cases** | **TNBC cases** | **Unknown subtype** |
| --- | --- | --- | --- | --- | --- | --- |
| Number of women, n | 7,844 | 3,784 | 1,480 | 264 | 500 | 1,816 |
| Number of deaths, n | 1,508 | 440 | 249 | 50 | 126 | 643 |
| Number of breast cancer-specific deaths, n | 721 | 163 | 113 | 33 | 81 | 331 |
| Age at diagnosis, mean ± SD | 63.2 ± 0.08 | 64.8 ± 0.11 | 64.6 ± 0.18 | 63.1 ± 0.45 | 64.0 ± 0.32 | 58.8 ± 0.17 |
| Age at death from breast cancer, mean ± SD | 66.7 ± 0.29 | 69.1 ± 0.60 | 68.3 ± 0.73 | 66.5 ± 1.56 | 66.8 ± 0.87 | 65.0 ± 0.41 |
| Tumor stage, n (%)  I  II  III  IV  Unknown | 4,191 (53.4)  2,082 (26.5)  453 (5.8)  206 (2.6)  912 (11.6) | 2,270 (60.0)  949 (25.1)  192 (5.1)  51 (1.4)  322 (8.5) | 781 (52.8)  418 (28.4)  99 (6.7)  33 (2.2)  149 (10.1) | 92 (34.9)  84 (31.8)  40 (15.2)  10 (3.8)  38 (14.4) | 217 (43.4)  148 (29.6)  42 (8.4)  20 (4.0)  73 (14.6) | 831 (45.8)  483 (26.6)  80 (4.4)  92 (5.1)  330 (18.2) |
| Tumor grade, n (%)  I  II  III  Unknown | 1,504 (19.2)  2,730 (34.8)  1,560 (19.9)  2,050 (26.1) | 937 (24.8)  1,443 (38.1)  436 (11.5)  968 (25.6) | 204 (13.8)  559 (37.8)  339 (22.9)  378 (25.5) | 3 (1.1)  72 (27.3)  155 (58.7)  34 (12.9) | 6 (1.2)  86 (17.2)  303 (60.6)  105 (21.0) | 354 (19.5)  570 (31.4)  327 (18.0)  565 (31.1) |
| Recent mammography screening (< 2 yrs) prior to diagnosis | 5,213 (66.5) | 2,716 (71.8) | 1,035 (69.9) | 181 (68.6) | 318 (63.6) | 963 (53.0) |
| Abbreviations: HER2: human epidermal growth factor receptor 2; TNBC: triple-negative breast cancer | | | | | | |

**Supplementary table 2.** Descriptives of cases according to MHT use at study entry

|  | **MHT use at study entry** | | | |
| --- | --- | --- | --- | --- |
|  | **Never MHT** | **Ever EPT use** | **Ever ET use only^1^** | **Ever unknown type** |
|  | Mean ± SD or n (%) | | | |
| Number of women, n (%) | 4,297 (54.8) | 2,599 (33.1) | 262 (3.3) | 686 (8.8) |
| Age at study entry (yrs) | 53.7 ± 0.08 | 53.1 ± 0.10 | 52.9 ± 0.35 | 51.6 ± 0.25 |
| Age at menarche (yrs) | 13.3 ± 0.02 | 13.2 ± 0.03 | 13.2 ± 0.09 | 13.3 ± 0.06 |
| Age at menopause (yrs) | 49.7 ± 0.09 | 49.8 ± 0.10 | 46.7 ± 0.38 | 48.5 ± 0.25 |
| Age at first birth (yrs)^2^ | 24.5 ± 0.08 | 24.1 ± 0.09 | 24.3 ± 0.31 | 23.7 ± 0.18 |
| Parity | 2.2 ± 0.02 | 2.1 ± 0.02 | 1.9 ± 0.07 | 2.1 ± 0.05 |
| BMI (kg/m^2^) | 24.9 ± 0.06 | 24.3 ± 0.07 | 24.4 ± 0.21 | 24.3 ± 0.14 |
| Alcohol consumption (g/day) | 1.12 ± 0.13 | 1.31 ± 0.02 | 1.23 ± 0.05 | 1.16 ± 0.03 |
| Education  ≤ 9 yrs  10-12 yrs  13-16 yrs  ≥ 17 yrs | 894 (22.2)  1,373 (34.0)  1.165 (28.9)  605 (15.0) | 463 (18.9)  910 (37.2)  697 (28.5)  379 (15.5) | 44 (17.5)  84 (33.5)  89 (35.5)  34 (13.6) | 186 (29.5)  211 (33.4)  156 (24.7)  78 (12.4) |
| Family history of breast cancer  None  Mother and sister  Mother  Sister | 3,819 (88.9)  23 (0.5)  319 (7.4)  136 (3.2) | 2,338 (90.0)  16 (0.6)  173 (6.7)  72 (2.8) | 237 (90.5)  0 (0.0)  15 (5.7)  10 (3.8) | 619 (90.2)  3 (0.4)  48 (7.0)  16 (2.3) |
| Smoking status, n (%)  Never  Former  Current | 1,586 (37.2)  1,447 (34.0)  1,226 (28.8) | 760 (29.4)  976 (37.7)  853 (33.0) | 80 (30.8)  106 (40.8)  74 (28.5) | 210 (31.3)  228 (33.9)  234 (34.8) |
| Physical activity, n (%)  Low  Moderate  High | 1,052 (26.3)  2,333 (58.4)  610 (15.3) | 648 (26.4)  1,452 (59.2)  352 (14.4) | 64 (26.3)  127 (52.3)  52 (21.4) | 171 (28.4)  344 (57.1)  88 (14.6) |
| Oral contraceptive use, n (%)  Never  Ever | 1,860 (44.7)  2,299 (55.3) | 1,025 (40.1)  1,533 (59.9) | 112 (43.8)  144 (56.3) | 285 (42.9)  380 (57.1) |
| ^1^ Never EPT users  ^2^ Among parous women  Abbreviations: EPT: estrogen-progestin therapy; ET: estrogen therapy; MHT: menopausal hormone therapy | | | | |

**Supplementary Table 3**. MHT use at study entry and incidence by intrinsic-like subtypes – age-adjusted analyses

|  | **Breast cancer overall**  **(n = 7,844)** | | **Luminal A-like**  **(n = 3,784)** | | **Luminal B-like**  **(n = 1,480)** | | **HER2+**  **(n = 264)** | | **TNBC**  **(n = 500)** | |
| --- | --- | --- | --- | --- | --- | --- | --- | --- | --- | --- |
|  | **n** | **HR (95% CI)** | **n** | **HR (95% CI)** | **n** | **HR (95% CI)** | **n** | **HR (95% CI)** | **n** | **HR (95% CI)** |
| **MHT use overall**  Never use  Ever use  Current  Former  Duration  < 5 yrs  ≥ 5 yrs  Per 1 yr | 4,297  3,547  2,782  765  2,250  1,243  7,790 | Ref.  1.25 (1.19-1.31)  1.36 (1.29-1.42)  0.97 (0.90-1.05)  1.16 (1.11-1.23)  1.43 (1.34-1.53)  1.04 (1.03-1.04) | 2,113  1,671  1,310  361  984  656  3,753 | Ref.  1.19 (1.12-1.27)  1.32 (1.24-1.42)  0.87 (0.78-0.98)  1.06 (0.99-1.15)  1.44 (1.32-1.58)  1.04 (1.03-1.05) | 845  635  464  171  416  212  1,473 | Ref.  1.16 (1.04-1.28)  1.19 (1.07-1.34)  1.06 (0.90-1.25)  1.14 (1.02-1.29)  1.20 (1.03-1.39)  1.02 (1.00-1.03) | 155  109  77  32  78  30  263 | Ref.  1.08 (0.85-1.39)  1.06 (0.80-1.39)  1.15 (0.78-1.68)  1.14 (0.86-1.50)  0.97 (0.66-1.44)  1.00 (0.96-1.04) | 297  203  139  64  128  71  496 | Ref.  1.04 (0.87-1.25)  1.00 (0.81-1.22)  1.15 (0.88-1.51)  0.98 (0.79-1.20)  1.16 (0.89-1.50)  1.01 (0.98-1.04) |
| **EPT use**  Never use  Ever use  Current  Former  Duration  < 5 yrs  ≥ 5 yrs  Per 1 yr | 4,297  2,599  2,120  479  1,559  1,028  6,884 | Ref.  1.33 (1.27-1.40)  1.44 (1.37-1.52)  0.99 (0.90-1.09)  1.22 (1.15-1.30)  1.53 (1.43-1.64)  1.04 (1.04-1.05) | 2,113  1,248  1,012  236  688  553  3,354 | Ref.  1.29 (1.20-1.39)  1.43 (1.32-1.54)  0.92 (0.81-1.06)  1.13 (1.04-1.23)  1.57 (1.43-1.72)  1.05 (1.04-1.05) | 845  464  352  112  288  175  1,308 | Ref.  1.23 (1.10-1.38)  1.26 (1.11-1.43)  1.13 (0.93-1.38)  1.20 (1.05-1.37)  1.28 (1.08-1.50)  1.03 (1.01-1.05) | 155  82  58  24  56  25  236 | Ref.  1.19 (0.91-1.55)  1.11 (0.82-1.51)  1.41 (0.92-2.18)  1.24 (0.91-1.69)  1.05 (0.69-1.61)  1.02 (0.97-1.06) | 297  147  107  40  91  55  443 | Ref.  1.10 (0.90-1.34)  1.07 (0.86-1.34)  1.17 (0.84-1.63)  1.06 (0.83-1.34)  1.16 (0.87-1.55)  1.01 (0.98-1.04) |
| **ET use only**  Never use  Ever use  Current  Former  Duration  < 5 yrs  ≥ 5 yrs  Per 1 yr | 4,297  262  224  38  164  96  4,557 | Ref.  0.98 (0.87-1.11)  1.05 (0.92-1.21)  0.70 (0.51-0.97)  0.98 (0.84-1.15)  0.98 (0.80-1.20)  1.00 (0.98-1.02) | 2,113  122  102  20  78  43  2,234 | Ref.  0.92 (0.77-1.11)  0.99 (0.81-1.20)  0.69 (0.45-1.08)  0.98 (0.78-1.23)  0.83 (0.62-1.13)  0.98 (0.96-1.01) | 845  52  47  5  29  22  896 | Ref.  1.00 (0.76-1.33)  1.16 (0.86-1.55)  0.45 (0.19-1.07)  0.92 (0.64-1.34)  1.09 (0.72-1.67)  1.01 (0.97-1.05) | 155  12  9  3  8  4  167 | Ref.  1.27 (0.71-2.29)  1.19 (0.61-2.34)  1.57 (0.50-4.94)  1.35 (0.66-2.75)  1.16 (0.43-3.14)  0.99 (0.89-1.11) | 297  15  12  3  8  7  312 | Ref.  0.82 (0.49-1.37)  0.83 (0.46-1.47)  0.78 (0.25-2.44)  0.71 (0.35-1.43)  1.01 (0.48-2.14)  0.98 (0.91-1.06) |
| **Cumulative dose**  Never use  Estrogen (E2-equivalence)  < 5 g  5 - 10 g  > 10 g  Progestin (NETA-equivalence)  < 1 g  1 - 2 g  > 2 g  E2 dose < 5 g  NETA dose < 1 g  NETA dose ≥ 1 g  E2 dose ≥ 5 g  NETA dose < 1 g  NETA dose ≥ 1 g | 4,297  1,999  827  192 | Ref.  1.23 (1.17-1.30)  1.39 (1.29-1.49)  1.54 (1.33-1.78) | 2,113  948  399  103 | Ref.  1.20 (1.11-1.29)  1.33 (1.19-1.48)  1.62 (1.33-1.97) | 845  347  154  34 | Ref.  1.12 (0.98-1.26)  1.32 (1.11-1.57)  1.37 (0.97-1.93) | 155  69  22  5 | Ref.  1.20 (0.91-1.60)  1.05 (0.67-1.65)  1.13 (0.46-2.77) | 297  112  48  6 | Ref.  1.01 (0.81-1.26)  1.16 (0.85-1.58)  0.68 (0.30-1.53) |
|  | 1,411  695  608  1,306  439  93  862 | 1.22 (1.15-1.29)  1.37 (1.26-1.48)  1.66 (1.52-1.80)  1.22 (1.15-1.30)  1.47 (1.33-1.62)  1.25 (1.01-1.53)  1.50 (1.39-1.61) | 634  361  304  589  233  40  431 | 1.14 (1.04-1.24)  1.42 (1.27-1.59)  1.60 (1.42-1.81)  1.14 (1.04-1.25)  1.54 (1.34-1.76)  1.10 (0.80-1.50)  1.48 (1.33-1.64) | 257  112  107  237  66  18  153 | 1.17 (1.02-1.35)  1.13 (0.93-1.38)  1.44 (1.18-1.77)  1.17 (1.01-1.35)  1.11 (0.87-1.43)  1.27 (0.80-2.03)  1.35 (1.13-1.60) | 46  18  18  43  12  3  23 | 1.12 (0.80-1.56)  1.00 (0.61-1.64)  1.39 (0.85-2.27)  1.13 (0.81-1.59)  1.14 (0.63-2.05)  1.14 (0.36-3.57)  1.14 (0.74-1.78) | 92  33  24  79  18  11  39 | 1.18 (0.93-1.49)  0.94 (0.66-1.35)  0.92 (0.61-1.40)  1.10 (0.85-1.41)  0.87 (0.54-1.39)  2.17 (1.19-3.97)  0.97 (0.70-1.36) |
| Abbreviations: CI: confidence interval; EPT: estrogen-progestin therapy; ET: estrogen therapy; E2: estradiol; HER2: human epidermal growth factor receptor 2; HR: hazard ratio; MHT: menopausal hormone therapy; NETA: norethisterone acetate; TNBC: triple-negative breast cancer. | | | | | | | | | | |

**Supplementary Table 4**. MHT use at study entry and incidence by intrinsic-like subtypes – complete-case, MV-adjusted analyses

|  | **Breast cancer**  **(n = 6,680)** | | **Luminal A-like**  **(n = 3,259)** | | **Luminal B-like**  **(n = 1,275)** | | **HER2-enriched**  **(n = 227)** | | **TNBC**  **(n = 422)** | |
| --- | --- | --- | --- | --- | --- | --- | --- | --- | --- | --- |
|  | **n** | **HR (95% CI)^1^** | **n** | **HR (95% CI)^1^** | **n** | **HR (95% CI)^1^** | **n** | **HR (95% CI)^1^** | **n** | **HR (95% CI)^1^** |
| **MHT use overall**  Never use  Ever use  Current  Former  Duration  < 5 yrs  ≥ 5 yrs  Per 1 yr | 3,660  3,020  2,397  623  1,930  1,056  6,646 | Ref.  1.22 (1.16-1.28)  1.34 (1.28-1.42)  0.90 (0.83-0.98)  1.15 (1.09-1.22)  1.37 (1.28-1.47)  1.03 (1.02-1.04) | 1,827  1,432  1,128  304  850  563  3,240 | Ref.  1.14 (1.07-1.23)  1.29 (1.19-1.39)  0.81 (0.72-0.92)  1.04 (0.96-1.13)  1.34 (1.22-1.47)  1.03 (1.02-1.04) | 733  542  414  128  356  183  1,272 | Ref.  1.09 (0.98-1.23)  1.18 (1.04-1.33)  0.90 (0.74-1.08)  1.09 (0.96-1.24)  1.12 (0.95-1.32)  1.01 (1.00-1.03) | 135  92  66  26  67  24  226 | Ref.  1.02 (0.78-1.34)  1.01 (0.75-1.36)  1.05 (0.69-1.60)  1.09 (0.81-1.47)  0.86 (0.55-1.33)  0.99 (0.95-1.04) | 253  169  116  53  106  59  418 | Ref.  0.99 (0.82-1.21)  0.96 (0.77-1.20)  1.08 (0.80-1.46)  0.93 (0.74-1.17)  1.08 (0.81-1.44)  1.00 (0.97-1.03) |
| **ETP use**  Never use  Ever use  Current  Former  Duration  < 5 yrs  ≥ 5 yrs  Per 1 yr | 3,660  2,255  1,850  405  1,364  881  5,905 | Ref.  1.29 (1.23-1.36)  1.42 (1.35-1.51)  0.91 (0.82-1.01)  1.21 (1.13-1.29)  1.44 (1.34-1.56)  1.04 (1.03-1.04) | 1,827  1,090  881  209  603  482  2,912 | Ref.  1.23 (1.14-1.32)  1.37 (1.26-1.49)  0.85 (0.73-0.98)  1.09 (1.00-1.20)  1.44 (1.30-1.59)  1.04 (1.03-1.05) | 733  406  317  89  255  150  1,138 | Ref.  1.16 (1.02-1.31)  1.23 (1.07-1.40)  0.96 (0.76-1.20)  1.16 (1.00-1.34)  1.16 (0.97-1.38)  1.02 (1.00-1.04) | 135  69  50  19  48  20  203 | Ref.  1.07 (0.80-1.44)  1.04 (0.75-1.44)  1.17 (0.72-1.91)  1.14 (0.82-1.60)  0.89 (0.55-1.43)  1.01 (0.96-1.05) | 253  125  91  34  77  47  377 | Ref.  1.04 (0.84-1.30)  1.03 (0.81-1.31)  1.08 (0.75-1.55)  1.01 (0.78-1.31)  1.08 (0.79-1.48)  1.00 (0.97-1.04) |
| **ET use only**  Never use  Ever use  Current  Former  Duration  < 5 yrs  ≥ 5 yrs  Per 1 yr | 3,660  226  197  29  140  84  3,884 | Ref.  0.95 (0.83-1.09)  1.04 (0.90-1.20)  0.60 (0.42-0.86)  0.94 (0.79-1.11)  0.97 (0.78-1.21)  0.99 (0.97-1.02) | 1,827  108  91  17  69  38  1,934 | Ref.  0.89 (0.73-1.08)  0.96 (0.78-1.19)  0.63 (0.39-1.01)  0.94 (0.74-1.20)  0.80 (0.58-1.10)  0.98 (0.95-1.01) | 733  45  42  3  25  19  777 | Ref.  0.94 (0.69-1.27)  1.10 (0.81-1.52)  0.29 (0.09-0.91)  0.86 (0.57-1.28)  1.04 (0.66-1.64)  1.00 (0.95-1.04) | 135  11  9  2  7  4  146 | Ref.  1.28 (0.69-2.38)  1.32 (0.67-2.59)  1.15 (0.28-4.67)  1.29 (0.60-2.76)  1.29 (0.48-3.50)  1.00 (0.90-1.12) | 253  10  8  2  6  4  263 | Ref.  0.61 (0.33-1.15)  0.62 (0.31-1.26)  0.58 (0.14-2.33)  0.60 (0.27-1.34)  0.65 (0.24-1.75)  0.93 (0.82-1.04) |
| **Cumulative dose**  Never use  Estrogen (E2-equivalence)  < 5 g  5 - 10 g  > 10 g  Progestin (NETA-equivalence)  < 1 g  1 - 2 g  > 2 g  E2 dose < 5 g  NETA dose < 1 g  NETA dose ≥ 1 g  E2 dose ≥ 5 g  NETA dose < 1 g  NETA dose ≥ 1 g | 3,660  1,745  721  152 | Ref.  1.20 (1.14-1.28)  1.34 (1.24-1.46)  1.37 (1.17-1.62) | 1,827  838  352  80 | Ref.  1.23 (1.13-1.33)  1.43 (1.27-1.62)  1.59 (1.27-2.00) | 733  307  132  29 | Ref.  1.12 (0.98-1.29)  1.33 (1.10-1.62)  1.41 (0.97-2.05) | 135  57  19  5 | Ref.  1.20 (0.87-1.65)  1.20 (0.73-1.98)  1.60 (0.65-3.95) | 253  93  41  5 | Ref.  0.96 (0.75-1.23)  1.16 (0.82-1.64)  0.70 (0.29-1.70) |
|  | 1,232  605  519  1,143  382  79  740 | 1.19 (1.11-1.27)  1.34 (1.23-1.46)  1.62 (1.48-1.79)  1.19 (1.12-1.28)  1.47 (1.32-1.64)  1.14 (0.91-1.42)  1.45 (1.33-1.57) | 567  317  256  526  202  36  370 | 1.17 (1.06-1.28)  1.54 (1.36-1.74)  1.79 (1.56-2.05)  1.17 (1.06-1.29)  1.71 (1.47-1.98)  1.14 (0.82-1.59)  1.60 (1.42-1.80) | 226  99  93  209  59  15  133 | 1.16 (0.99-1.34)  1.18 (0.95-1.46)  1.57 (1.25-1.96)  1.15 (0.99-1.35)  1.22 (0.93-1.59)  1.17 (0.70-1.96)  1.40 (1.15-1.69) | 38  14  16  35  9  3  20 | 1.08 (0.75-1.57)  1.03 (0.58-1.81)  1.82 (1.05-3.13)  1.08 (0.74-1.58)  1.16 (0.59-2.31)  1.42 (0.45-4.50)  1.35 (0.83-2.21) | 77  29  21  68  15  8  35 | 1.13 (0.87-1.46)  0.98 (0.66-1.46)  1.01 (0.64-1.60)  1.07 (0.82-1.41)  0.88 (0.52-1.49)  1.80 (0.88-3.65)  1.06 (0.73-1.52) |
| ^1^ Adjusted for BMI, parity, age at first birth, age at menarche, family history, smoking, physical activity, education  Abbreviations: CI: confidence interval; ETP: estrogen-progestin therapy; ET: estrogen therapy; E2: estradiol; HER2: human epidermal growth factor receptor 2; HR: hazard ratio; MHT: menopausal hormone therapy; MV: multivariable; NETA: norethisterone acetate; TNBC: triple-negative breast cancer | | | | | | | | | | |

**Supplementary Table 5**. MHT use at study entry and breast cancer-specific mortality by intrinsic-like subtypes – complete-case dataset

|  | **Breast cancer overall** | | **Luminal A-like** | | **Luminal B-like** | | **HER2-enriched** | | **TNBC** | |
| --- | --- | --- | --- | --- | --- | --- | --- | --- | --- | --- |
|  | **n** | **HR (95% CI)** | **n** | **HR (95% CI)** | **n** | **HR (95% CI)** | **n** | **HR (95% CI)** | **n** | **HR (95% CI)** |
| **Age-adjusted**  **MHT use overall**  Never use  Ever use  Current  Former  Duration  < 5 yrs  ≥ 5 yrs  Per 1 yr | 721  392  329  268  61  220  104  716 | Ref.  1.26 (1.09-1.47)  1.46 (1.25-1.71)  0.79 (0.61-1.04)  1.29 (1.09-1.52)  1.22 (0.98-1.51)  1.02 (1.00-1.04) | 163  82  81  65  16  43  35  160 | Ref.  1.48 (1.09-2.02)  1.73 (1.24-2.40)  0.94 (0.55-1.61)  1.24 (0.85-1.79)  1.87 (1.26-2.79)  1.06 (1.02-1.10) | 113  64  49  39  10  31  17  112 | Ref.  1.13 (0.78-1.64)  1.29 (0.87-1.93)  0.76 (0.39-1.47)  1.10 (0.72-1.70)  1.15 (0.67-1.98)  1.02 (0.97-1.07) | 33  20  13  13  0  10  3  33 | Ref.  0.95 (0.47-1.93)  1.35 (0.66-2.74)  -  1.11 (0.51-2.39)  0.69 (0.20-2.33)  0.95 (0.83-1.08) | 81  54  27  16  11  16  11  81 | Ref.  0.74 (0.47-1.18)  0.63 (0.36-1.10)  1.02 (0.53-1.96)  0.68 (0.39-1.18)  0.91 (0.48-1.76)  0.99 (0.92-1.06) |
| **EPT use**  Never use  Ever use  Current  Former  Duration  < 5 yrs  ≥ 5 yrs  Per 1 yr | 392  237  208  29  152  84  628 | Ref.  1.33 (1.13-1.57)  1.58 (1.34-1-88)  0.62 (0.43-0.91)  1.37 (1.13-1.65)  1.27 (1.00-1.61)  1.02 (1.00-1.05) | 82  62  54  8  30  31  143 | Ref.  1.68 (1.21-2.35)  2.01 (1.43-2.85)  0.79 (0.38-1.64)  1.35 (0.89-2.06)  2.16 (1.42-3.27)  1.08 (1.04-1.12) | 64  37  31  6  23  14  101 | Ref.  1.25 (0.83-1.89)  1.44 (0.93-2.22)  0.75 (0.32-1.74)  1.27 (0.78-2.05)  1.23 (0.69-2.20)  1.03 (0.98-1.09) | 20  11  11  0  9  2  31 | Ref.  1.20 (0.57-2.53)  1.62 (0.77-3.40)  -  1.57 (0.71-3.48)  0.60 (0.14-2.57)  0.94 (0.81-1.10) | 54  20  14  6  11  9  74 | Ref.  0.81 (0.48-1.35)  0.76 (0.42-1.38)  0.92 (0.39-2.14)  0.71 (0.37-1.37)  0.97 (0.48-1.97)  0.99 (0.92-1.07) |
| **MV-adjusted^1^**  **MHT use overall**  Never use  Ever use  Current  Former  Duration  < 5 yrs  ≥ 5 yrs  Per 1 yr | 585  309  276  226  50  195  79  583 | Ref.  1.30 (1.10-1.53)  1.51 (1.27-1.80)  0.80 (0.60-1.09)  1.40 (1.16-1.67)  1.13 (0.88-1.45)  1.01 (0.99-1.04) | 128  65  63  49  14  36  25  126 | Ref.  1.44 (1.01-2.05)  1.66 (1.14-2.42)  0.99 (0.55-1.77)  1.30 (0.86-1.97)  1.62 (1.01-2.58)  1.05 (1.01-1.09) | 92  50  42  35  7  29  13  92 | Ref.  1.16 (0.76-1.75)  1.41 (0.91-2.19)  0.61 (0.28-1.35)  1.27 (0.80-2.02)  0.99 (0.53-1.84)  1.00 (0.95-1.06) | 28  19  10  10  0  9  1  29 | Ref.  0.83 (0.37-1.82)  1.18 (0.53-2.61)  -  1.10 (0.49-2.51)  0.27 (0.04-2.01)  0.89 (0.73-1.07) | 67  44  23  13  10  14  9  67 | Ref.  0.74 (0.45-1.24)  0.59 (0.32-1.11)  1.10 (0.55-2.21)  0.69 (0.38-1.28)  0.87 (0.42-1.80)  0.98 (0.91-1.06) |
| **EPT use**  Never use  Ever use  Current  Former  Duration  < 5 yrs  ≥ 5 yrs  Per 1 yr | 309  200  174  26  137  63  509 | Ref.  1.35 (1.13-1.61)  1.60 (1.32-1.93)  0.66 (0.44-0.99)  1.48 (1.21-1.81)  1.14 (0.86-1.50)  1.01 (0.99-1.04) | 65  47  39  8  25  22  112 | Ref.  1.56 (1.06-2.28)  1.83 (1.22-2.74)  0.91 (0.43-1.90)  1.40 (0.88-2.23)  1.81 (1.11-2.96)  1.07 (1.02-1.11) | 50  33  28  5  23  10  83 | Ref.  1.29 (0.82-2.01)  1.54 (0.96-2.46)  0.67 (0.27-1.69)  1.51 (0.92-2.49)  0.96 (0.48-1.90)  1.01 (0.95-1.07) | 19  9  9  0  8  1  28 | Ref.  1.06 (0.47-2.40)  1.45 (0.64-3.30)  -  1.46 (0.62-3.44)  0.33 (0.04-2.53)  0.91 (0.75-1.10) | 44  18  12  6  10  8  62 | Ref.  0.83 (0.48-1.45)  0.75 (0.39-1.44)  1.03 (0.44-2.45)  0.75 (0.37-1.49)  0.98 (0.45-2.09)  1.00 (0.92-1.08) |
| ^1^ Adjusted for BMI, parity, age at first birth, age at menarche, family history, smoking, physical activity, education  Abbreviations: CI: confidence interval; EPT: estrogen-progestin therapy; HER2: human epidermal growth factor receptor 2; HR: hazard ratio; MHT: menopausal hormone therapy; TNBC: triple-negative breast cancer | | | | | | | | | | |

**Supplementary Table 6**. MHT use at study entry and 10-year survival by intrinsic-like subtypes – complete-case analyses

|  | **Breast cancer overall** | | **Luminal A-like** | | **Luminal B-like** | | **HER2+** | | **TNBC** | |
| --- | --- | --- | --- | --- | --- | --- | --- | --- | --- | --- |
|  | **n** | **HR (95% CI)^1^** | **n** | **HR (95% CI)^1^** | **n** | **HR (95% CI)^1^** | **n** | **HR (95% CI)^1^** | **n** | **HR (95% CI)^1^** |
| **Age-adjusted**  **MHT use overall**  Never use  Ever use  Current  Former  Duration  < 5 yrs  ≥ 5 yrs  Per 1 yr | 634  356  278  226  52  181  93  630 | Ref.  0.92 (0.79-1.08)  0.94 (0.79-1.11)  0.84 (0.63-1.13)  0.93 (0.77-1.11)  0.91 (0.72-1.15)  0.99 (0.97-1.01) | 148  76  72  58  14  38  32  146 | Ref.  1.11 (0.80-1.54)  1.15 (0.82-1.63)  0.96 (0.54-1.71)  0.96 (0.65-1.43)  1.33 (0.87-2.03)  1.03 (0.99-1.07) | 104  62  42  32  10  26  15  103 | Ref.  0.74 (0.50-1.10)  0.75 (0.49-1.16)  0.84 (0.43-1.65)  0.76 (0.48-1.21)  0.76 (0.43-1.35)  0.98 (0.93-1.04) | 32  19  13  13  0  10  3  32 | Ref.  0.85 (0.42-1.72)  1.06 (0.52-2.16)  -  1.00 (0.46-2.18)  0.58 (0.17-1.97)  0.92 (0.81-1.06) | 81  54  27  16  11  16  11  81 | Ref.  0.57 (0.36-0.91)  0.43 (0.24-0.75)  1.12 (0.58-2.16)  0.53 (0.30-0.93)  0.66 (0.34-1.27)  0.95 (0.88-1.03) |
| **EPT use**  Never use  Ever use  Current  Former  Duration  < 5 yrs  ≥ 5 yrs  Per 1 yr | 356  201  175  26  126  74  556 | Ref.  0.91 (0.77-1.09)  0.96 (0.80-1.15)  0.69 (0.46-1.03)  0.93 (0.76-1.15)  0.87 (0.68-1.13)  0.99 (0.96-1.01) | 76  54  47  7  25  28  129 | Ref.  1.14 (0.80-1.63)  1.22 (0.85-1.77)  0.79 (0.36-1.72)  0.92 (0.58-1.45)  1.42 (0.92-2.21)  1.04 (1.00-1.08) | 62  31  25  6  19  12  93 | Ref.  0.77 (0.50-1.20)  0.76 (0.48-1.22)  0.81 (0.35-1.88)  0.80 (0.48-1.34)  0.73 (0.39-1.37)  0.99 (0.93-1.05) | 19  11  11  0  9  2  30 | Ref.  1.00 (0.47-2.12)  1.20 (0.57-2.53)  -  1.35 (0.61-3.00)  0.47 (0.11-2.03)  0.92 (0.79-1.07) | 54  20  14  6  11  9  74 | Ref.  0.57 (0.34-0.96)  0.49 (0.27-0.88)  0.99 (0.42-2.33)  0.53 (0.28-1.02)  0.65 (0.32-1.33)  0.95 (0.88-1.03) |
| **MV-adjusted^2^**  **MHT use overall**  Never use  Ever use  Current  Former  Duration  < 5 yrs  ≥ 5 yrs  Per 1 yr | 515  280  235  193  42  162  72  514 | Ref.  1.00 (0.84-1.19)  1.02 (0.85-1.23)  0.90 (0.65-1.25)  1.05 (0.86-1.28)  0.91 (0.70-1.19)  0.99 (0.96-1.02) | 116  60  56  44  12  32  23  115 | Ref.  1.16 (0.80-1.69)  1.19 (0.80-1.77)  1.08 (0.58-2.03)  1.08 (0.70-1.68)  1.28 (0.78-2.11)  1.03 (0.98-1.08) | 85  48  37  30  7  25  12  85 | Ref.  0.87 (0.56-1.36)  0.91 (0.57-1.46)  0.74 (0.33-1.66)  0.97 (0.59-1.58)  0.75 (0.39-1.43)  0.98 (0.92-1.04) | 27  17  10  10  0  9  1  27 | Ref.  0.78 (0.35-1.73)  0.97 (0.44-2.18)  -  1.00 (0.44-2.30)  0.26 (0.03-2.01)  0.88 (0.72-1.07) | 67  44  23  13  10  14  9  67 | Ref.  0.59 (0.35-0.99)  0.41 (0.22-0.78)  1.30 (0.64-2.61)  0.56 (0.31-1.04)  0.66 (0.32-1.38)  0.95 (0.88-1.04) |
| **EPT use**  Never use  Ever use  Current  Former  Duration  < 5 yrs  ≥ 5 yrs  Per 1 yr | 280  172  148  24  115  57  452 | Ref.  0.99 (0.81-1.20)  1.02 (0.83-1.25)  0.82 (0.54-1.25)  1.07 (0.86-1.33)  0.86 (0.65-1.15)  0.99 (0.96-1.02) | 60  41  34  7  21  20  101 | Ref.  1.14 (0.76-1.72)  1.19 (0.77-1.83)  0.97 (0.44-2.13)  1.01 (0.61-1.67)  1.34 (0.80-2.26)  1.04 (0.99-1.09) | 48  28  23  5  19  9  76 | Ref.  0.87 (0.54-1.41)  0.89 (0.54-1.48)  0.80 (0.31-2.02)  1.04 (0.60-1.78)  0.66 (0.32-1.37)  0.98 (0.91-1.05) | 17  9  9  0  8  1  26 | Ref.  0.96 (0.42-2.20)  1.15 (0.50-2.65)  -  1.29 (0.54-3.04)  0.32 (0.04-2.42)  0.89 (0.73-1.09) | 44  18  12  6  10  8  62 | Ref.  0.62 (0.36-1.09)  0.50 (0.26-0.95)  1.24 (0.52-2.95)  0.58 (0.29-1.15)  0.70 (0.33-1.52)  0.97 (0.89-1.06) |
| ^1^ HRs of breast cancer-specific deaths  ^2^ Adjusted for BMI, parity, age at first birth, age at menarche, family history, smoking, physical activity, education  Abbreviations: CI: confidence interval; EPT: estrogen-progestin therapy; HER2: human epidermal growth factor receptor 2; HR: hazard ratio; MHT: menopausal hormone therapy; TNBC: triple-negative breast cancer | | | | | | | | | | |

# Supplementary figures

**Supplementary Figure 1**. Flow chart of study sample


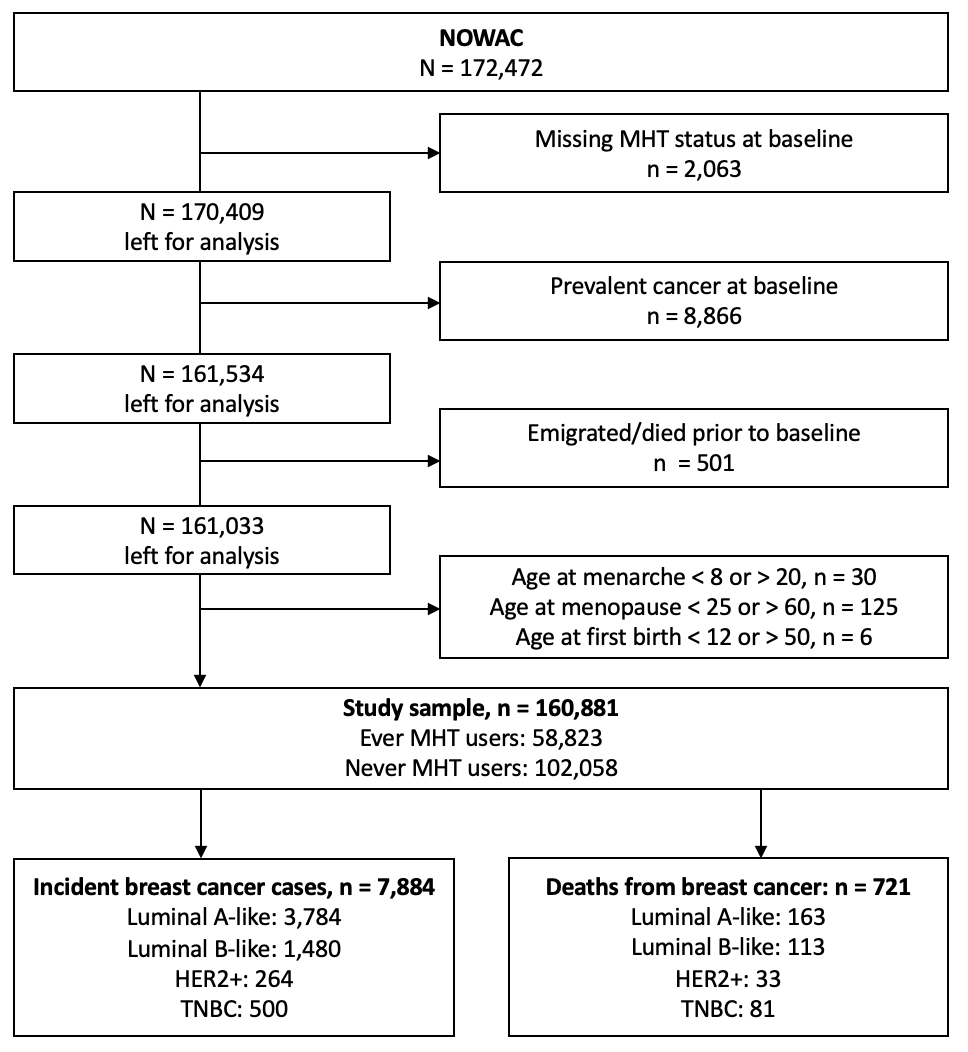


**Supplementary Figure 2.** Directed acyclic graph on the assumed relations between MHT use and incidence of postmenopausal breast cancer


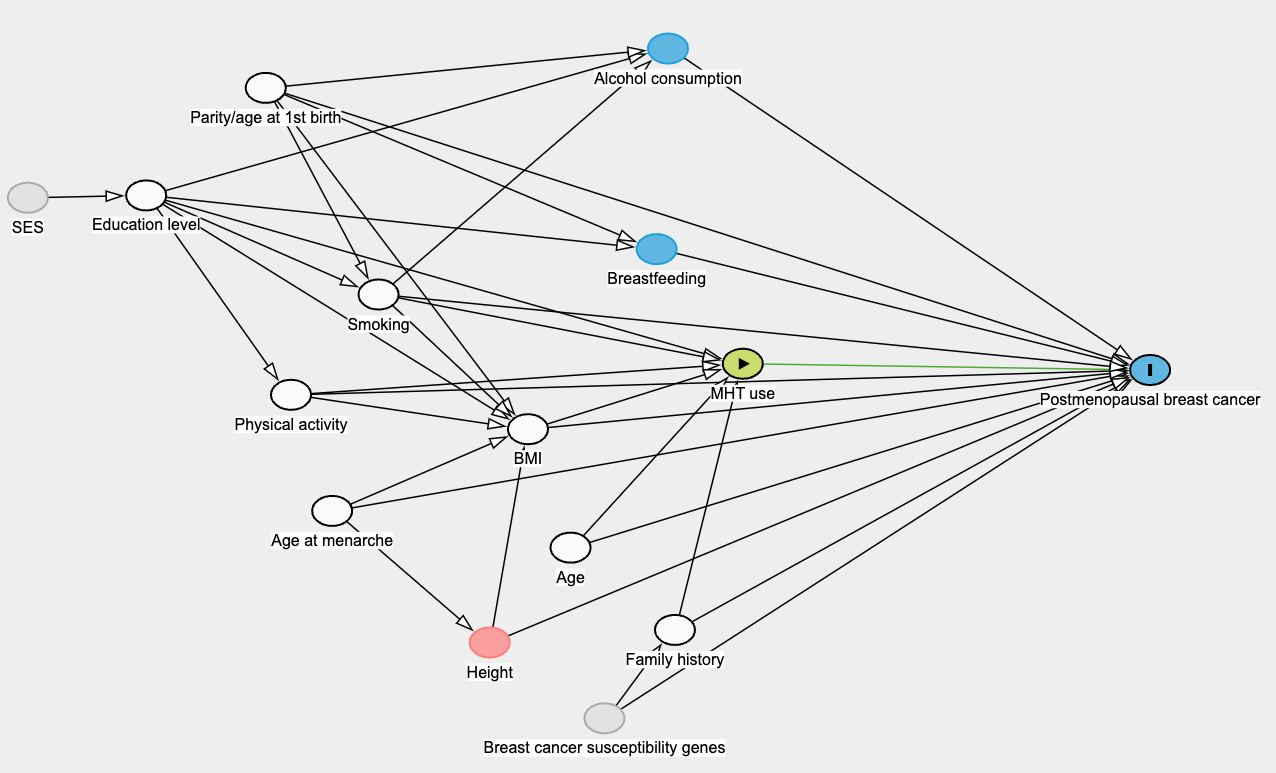


Directed acyclic graph for the effect of MHT use and breast cancer incidence. Created from <https://dagitty.net>. Minimal sufficient adjustment set, depicted in white, included age, age at menarche, BMI, education level, family history of breast cancer, parity/age at first birth, physical activity and smoking.

##

**Supplementary Figure 3**. Directed acyclic graph on the assumed relations between MHT use and mortality of postmenopausal breast cancer


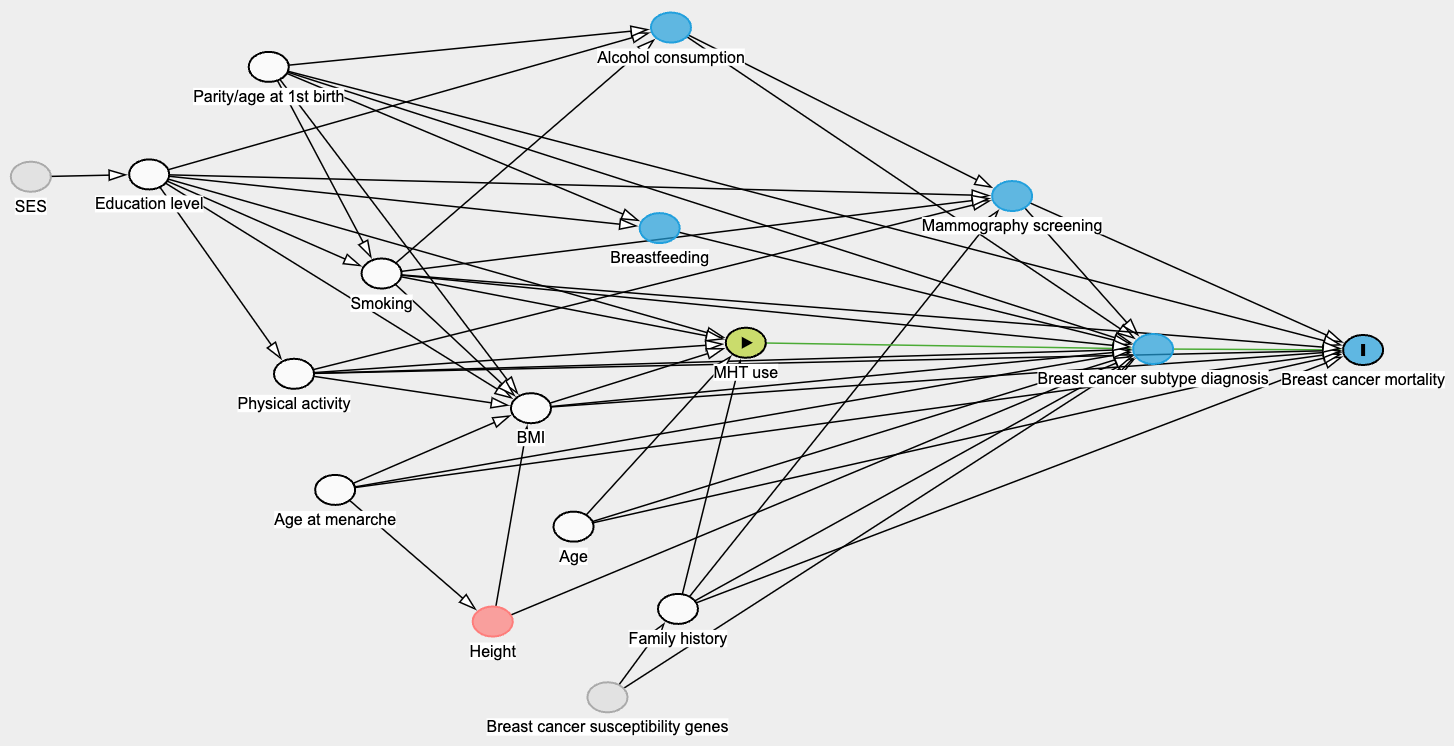


Directed acyclic graph for the effect of MHT use and breast cancer mortality. Created from <https://dagitty.net>. Minimal sufficient adjustment set, depicted in white, included age, age at menarche, BMI, education level, family history of breast cancer, parity/age at first birth, physical activity and smoking.

##

**Supplementary Figure 4**. Directed acyclic graph on the assumed relations between MHT use and survival of postmenopausal breast cancer


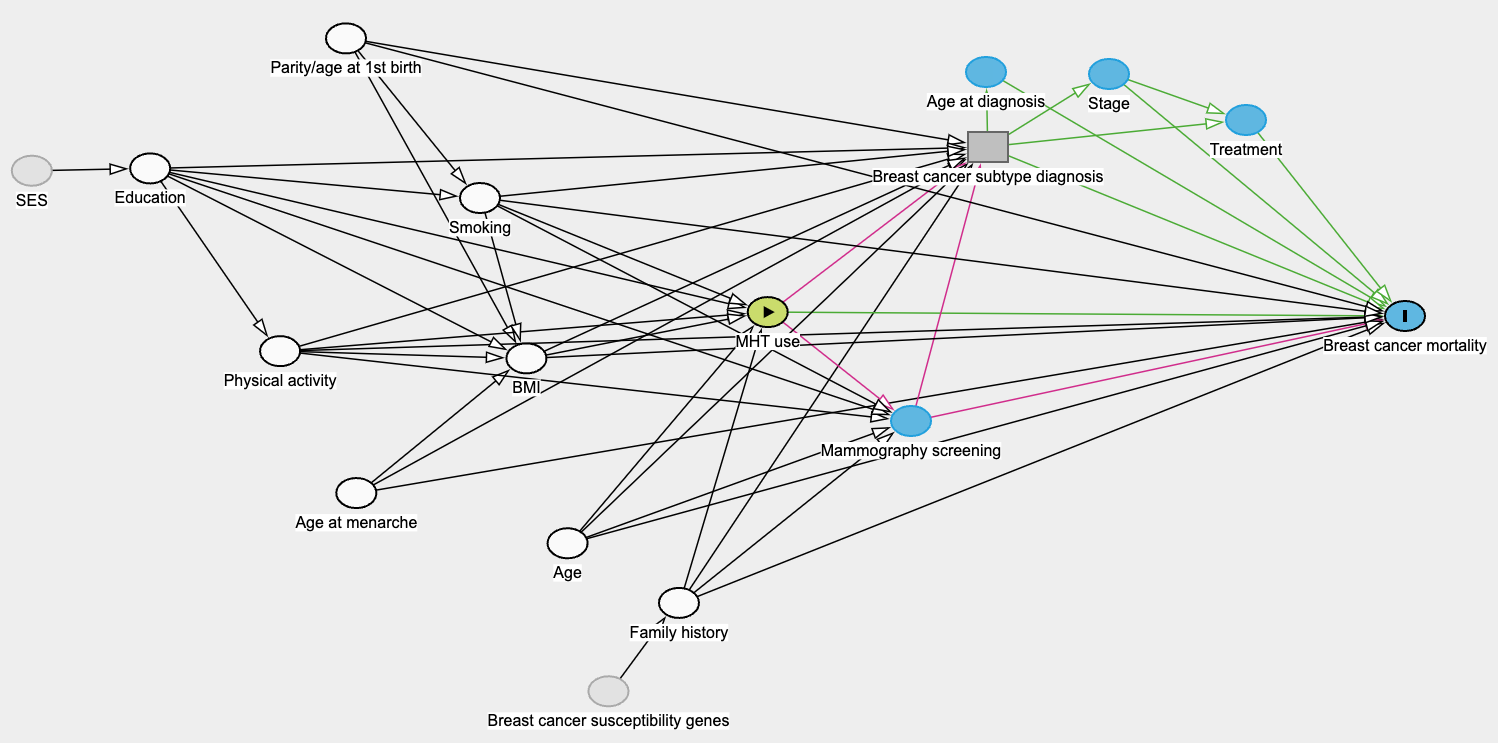


Directed acyclic graph for the effect of MHT use and breast cancer survival. Created from <https://dagitty.net>. Adjustments were made for variables depicted in white: age, age at menarche, BMI, education level, family history of breast cancer, parity/age at first birth, physical activity and smoking.
